# A Multi-Agent Framework for Packet Routing in Wireless Sensor Networks

**DOI:** 10.3390/s150510026

**Published:** 2015-04-28

**Authors:** Dayon Ye, Minji Zhang, Yu Yang

**Affiliations:** 1 School of Software and Electrical Engineering, Swinburne University of Technology, Melbourne 3122, Australia; E-Mail: yyang@swin.edu.au; 2 School of Computer Science and Software Engineering, University of Wollongong, Wollongong 2522, Australia; E-Mail: minjie@uow.edu.au

**Keywords:** multi-agent systems, wireless sensor networks, packet routing

## Abstract

Wireless sensor networks (WSNs) have been widely investigated in recent years. One of the fundamental issues in WSNs is packet routing, because in many application domains, packets have to be routed from source nodes to destination nodes as soon and as energy efficiently as possible. To address this issue, a large number of routing approaches have been proposed. Although every existing routing approach has advantages, they also have some disadvantages. In this paper, a multi-agent framework is proposed that can assist existing routing approaches to improve their routing performance. This framework enables each sensor node to build a cooperative neighbour set based on past routing experience. Such cooperative neighbours, in turn, can help the sensor to effectively relay packets in the future. This framework is independent of existing routing approaches and can be used to assist many existing routing approaches. Simulation results demonstrate the good performance of this framework in terms of four metrics: average delivery latency, successful delivery ratio, number of live nodes and total sensing coverage.

## 1. Introduction

Due to recent technological advances, the manufacturing of small, low-power, low-cost and highly integrated sensors has become technically and economically feasible. These sensors are generally equipped with sensing, data processing and communicating components. Thus, such sensors can be used to measure ambient conditions in the environment surrounding them and then transform these measurements into signals. The signals can be processed further to reveal some properties about objects located in the vicinity of the sensors. The sensors then send such collected data, usually via a radio transmitter, to a command centre (also known as a sink or a base station) either directly or via several relaying sensors. A large number of these sensors can be networked in many applications that require unattended operations, hence producing a WSN. Currently, there are various applications of WSNs. These applications include battlefield surveillance [1], video capture, processing and communication [2,3], *etc*. Typically, WSNs contain hundreds or thousands of sensors, and these sensors have the ability to communicate with each other. Thus, packet routing is an important issue in WSNs. In addition, the routing issue in WSNs is more challenging than in other types of wireless networks, e.g., mobile *ad hoc* networks or cellular networks [4], because the power energy, storage capability and processing capacity of each sensor are usually constrained. Moreover, unlike general computer networks, where the destination of data is variable, in WSNs, the destination of nearly all data is a sink node. Therefore, designing routing approaches in such sensor networks needs careful resource management.

Over the last decade, many approaches have been proposed in order to solve the routing problem in WSNs [5,6]. These approaches have taken into account the inherent features of WSNs along with particular applications and architecture requirements. It is not a simple task to find and maintain routes in WSNs due to energy constraints and sensor failures. To mitigate energy consumption, existing routing approaches proposed for WSNs have employed some well-known routing techniques from other wireless networks and special techniques in WSNs, such as data aggregation, clustering, different node role assignment, *etc*. According to Al-Karaki and Kamal [7], almost all of the routing approaches can be classified into three major categories: flat routing approaches, hierarchical routing approaches and location-based (also known as geographic) routing approaches, although there are a few exceptional ones based on network flow or quality of service (QoS) awareness [8]. Routing approaches belonging to each of the three categories have advantages and disadvantages. In flat routing approaches, all sensors play the same role, and each sensor could be a source node or a relay node. Hence, the routing delay can be minimised, if the routing algorithm is carefully designed. The energy consumption is an outstanding problem, however, compared with the routing approaches belonging to other two categories, because sensors lack the knowledge of the global network, and thus, broadcasting is often required or data have to be repeatedly transferred in the network. Hierarchical routing approaches aim to cluster sensor nodes so that cluster heads can aggregate and reduce data to be transmitted to the sink node in order to save energy. Because packet transmission is the most energy expensive task [9], the reduction of packets is vital for the overall network. However, the packet routing delay will be lengthened, as cluster heads have to wait for a period for sensors in their clusters to transfer packets to them for aggregation. Location-based routing approaches use the position information to relay the packets to the desired regions rather than the whole network. Location-based routing approaches have good scalability, since the information of the whole network is not required. Such location-based approaches, however, often need a device, such as a GPS, to provide the necessary position information. Furthermore, the cost and weight of a sensor will be increased if each sensor is equipped with such a device. Although several researchers have suggested using a virtual coordinate to provide the position information [10], this would incur extra energy consumption due to the communication process, which is necessary for acquiring such position information. In addition, to obtain such virtual coordinate-based position information, global information has to be used, e.g., the maximum ID among all sensors with the maximum hop distance from a specific node.

As can be seen from the above, since all existing routing approaches have some disadvantages, it is more important to develop an assistant tool to enhance existing routing approaches than to develop a new routing approach. To the best of our knowledge, this paper is the first one to propose a multi-agent framework that can be used to assist and enhance many existing routing approaches. The merits of this framework include that: (i) it is independent of individual existing routing approaches, so it can assist many existing routing approaches and enhance their performance; (ii) it is decentralised and operated based only on local information, and it does not need to change the physical network topology; and (iii) it does not only suit static WSNs, but also works well in mobile and open WSNs, where sensors are mobile and new sensors may join or existing sensors may leave the network.

The rest of the paper is organised as follows. In the next section, a detailed literature review is provided, which includes routing approaches belonging to the aforementioned three categories, especially those approaches developed after the publication of the survey papers in [4,7]. Then, the proposed framework is described in detail in Section 3. A simulation regarding the performance of the proposed framework is given in Section 4. Finally, this paper is concluded in Section 5.

## 2. Literature Review

As the routing issue is one of the most significant issues in WSNs, there is a large number of published prominent studies that focus on this issue. Akkaya and Younis [4] and Al-Karaki and Kamal [7] provided a thorough survey on routing approaches nearly at the same time. Thus, in this section, our review focuses primarily on the routing approaches developed after their survey papers were published.

### 2.1. Flat Routing Approaches

The first category of routing approaches is multi-hop flat routing. In such routing approaches, each sensor node typically plays the same role, and sensor nodes collaborate to perform sensing tasks.

Flooding and gossiping [11] are two classical approaches to relay packets in sensor networks without the need for any routing algorithms or topology maintenance. In flooding, each sensor that receives a packet broadcasts the packet to all of its neighbours, and this process continues until the packet arrives at the destination or the maximum number of hops for the packet is reached. On the other hand, gossiping is a slightly enhanced version of flooding where the receiving node sends the packet to a randomly-selected neighbour, which picks another random neighbour to forward the packet to, and so on, until the destination or the maximum hop is reached.

Heinzelman *et al.* [12,13] proposed a family of adaptive protocols, called sensor protocols for information via negotiation (SPIN). The SPIN family is designed to address the deficiencies of classical routing approaches, such as flooding and gossiping, which waste much energy and bandwidth when sending extra and unnecessary copies of packets by sensors that cover overlapping areas. SPIN consists of three steps. First, when a sensor has a data packet to transfer, it broadcasts the metadata of the data packet to its neighbours. The size of the meta packets is much smaller than that of the data packet. Then, if any neighbours are interested in the data packet, they will send back a request message to the sensor. Once the sensor receives the request message, it will transfer the data packet to those neighbours that are interested in that packet.

Rogers *et al.* [14] devised an energy-aware self-organised routing algorithm for WSNs. Their algorithm enables individual sensors to follow locally selfish strategies, which, in turn, result in a framework of a routing network with desirable global properties. In addition, the algorithm can adaptively deal with the changing sensor numbers and network topology.

Beraldi *et al.* [15] studied the lowest latency path problem and then devised a forwarding protocol based on biased random walks, where sensors use only local information about their neighbours and their next active period to make forwarding decisions. Specifically, their protocol, lukewarm potato, is a compromise between the shortest path forwarding and hot potato forwarding. Hot potato forwarding is a greedy approach to the fast propagation of a message toward the sink: a node, upon receiving a message, forwards the message to the neighbour that is the first to become active.

Quang and Kim [16] devised a gradient routing algorithm that uses two-hop information for industrial WSNs to enhance real-time performance with energy efficiency. The routing algorithm is based on the number of hops to the sink instead of distance. Specifically, the routing algorithm applied two-hop velocity-based routing [17] to a gradient-based WSNs so as to reduce both energy consumption and end-to-end delay.

Niu *et al.* [18] introduced a routing enhancement scheme. Their scheme first finds a robust guide path during the route discovery phase. Then, along this guide path, data packets are greedily forwarded toward the destination through nodes' cooperation without utilising location information. The forwarding procedure is based on the priorities of forwarding candidates, where nodes with the highest priorities will be picked up to forward data packets.

### 2.2. Hierarchical Routing Approaches

Hierarchical or cluster-based routing approaches, originally proposed in wired networks, are well-known techniques with special advantages related to efficient communication. The concept of hierarchical routing is also utilised to perform energy-efficient routing in WSNs. In a hierarchical architecture, high-energy sensors can be used to process and transmit information, whereas low-energy sensors can be employed to perform sensing tasks in the proximity of the target. Hierarchical routing is usually a two-layer routing approach, where one layer is composed of cluster heads and the other layer consists of cluster members. Cluster members are responsible for sensing tasks, while cluster heads are used to aggregate packets and transmit the aggregated packets to the sink node. Since the size of aggregated packets is much less than that of raw packets, the energy consumption used for packet transmission can be significantly reduced, and thus, the overall network lifetime can be prolonged.

The most famous hierarchical routing protocol is LEACH (low-energy adaptive clustering hierarchy) [19]. LEACH randomly selects a few sensor nodes as cluster heads and rotates this role to evenly distribute the energy load among the sensors in the network. In LEACH, cluster heads compress packets arriving from cluster members that belong to the respective clusters. Cluster heads then send aggregate packets to a sink in order to reduce the number of packets that must be transmitted to the sink.

In [20], an enhancement over the LEACH protocol was proposed, *i.e.*, power-efficient gathering in sensor information systems (PEGASIS). PEGASIS is a near optimal chain-based protocol. The basic idea is that in order to extend network lifetime, sensor nodes need to communicate only with their closest neighbours, and these neighbours take turns in communicating with the sink. Unlike LEACH, PEGASIS avoids cluster formation and uses only one sensor node in a chain instead of multiple nodes to transmit the sink.

Tsai [21] presented a coverage-preserving routing protocol that was a modified version of LEACH. Unlike most routing protocols, the aim of his protocol is to preserve sensing coverage when some sensor nodes are no longer available, because, for example, their energy is used up. Tsai considered that simply increasing the lifetime of sensor nodes might not automatically achieve good sensing coverage. Thus, unlike LEACH, Tsai's protocol considered not only energy consumption, but also sensing coverage when clusters were formed.

Valentini *et al.* [22] devised a non-dominated algorithm to improve a simple hybrid routing protocol (SHRP) [23] in choosing the best route towards the sink node. Their algorithm allows simultaneous analysis of energy- and latency-related metrics to generate a Pareto-optimal solution, which has better time convergence and reliability than SHRP.

Mottola and Picco [24] developed an adaptive energy-aware routing protocol that was expressly designed for many-to-many communication, *i.e.*, simultaneously routing from multiple sources to multiple sinks. To increase network lifetime, their protocol minimises the number of nodes involved in many-to-many routing and balances forwarding load, reduces the amount of redundant information flowing in the network and decreases contention on the wireless medium and packet collisions.

### 2.3. Location-Based Routing Approaches

In this kind of routing, also known as geographic routing, sensor nodes are addressed in terms of their locations. The distance between neighbouring nodes can be estimated on the basis of incoming signal strength. The relative coordinates of neighbouring nodes can be obtained by exchanging information between neighbours. Alternatively, the location of sensor nodes may be achieved directly by communicating with a satellite using GPS if each node is equipped with a small low-power GPS receiver.

GAF (geographic adaptive fidelity) [25] is an energy-aware location-based routing algorithm designed primarily for mobile *ad hoc* networks, but may be applicable to WSNs, as well. In GAF, the network area is first divided into fixed zones. These zones then form a virtual grid. Inside each zone, nodes collaborate with each other to play different roles, and each node uses its GPS-indicated location to associate itself with a point on the virtual grid.

Yu *et al.*'s protocol [26], *i.e.*, GEAR (geographic and energy-aware routing), uses energy-aware and geographically-informed neighbour selection heuristics to route a packet toward the destination region. The key idea is to restrict the number of messages in directed diffusion by considering only a certain region rather than sending the messages to the whole network. Thus, the energy for transmission can be conserved.

Huang *et al.* [27] presented an energy-aware and interference-sensitive geographic routing protocol that focuses on minimising the total network energy consumption and reducing interference. Their routing protocol adaptively uses an anchor list to guide packet delivery and selects the minimum-interference link from the energy-optimal relay region for packet delivery.

Awad *et al.* [28] proposed the virtual cord protocol (VCP), which exploits virtual coordinates to provide efficient and failure-tolerant routing and packet management in sensor networks. Specifically, VCP uses two mechanisms to find paths to nodes and associated packet items. First, it relies on the virtual cord that always provides a path toward the destination. Second, locally available neighbourhood information is exploited for greedy routing.

Petrioli *et al.* [29] presented a geographic routing protocol that considered three important, but often neglected design challenges: routing around connectivity holes, resilience to localisation errors and efficient relay selection. In addition, their protocol integrates awake/asleep schedules, MAC, routing, traffic, load balancing and back-to-back transmissions. During the forwarding procedure, a sender checks the availability of its awake neighbouring nodes and channels. Then, the sender chooses the best relaying node based on its neighbouring nodes' forwarding performance. If several nodes have the same performance, positive geographic advancement toward the sink is used to discriminate among them. Such joint optimisation can make the protocol robust and realistic.

After reviewing related work, it can be seen that each approach has its own strengths and limitations. We propose, therefore, that it is important to devise a framework that can be applied to many existing routing approaches in order to improve their performance. This paper proposes such a framework, which will be described in detail in the next section.

## 3. The Multi-Agent Framework

**Figure 1:**
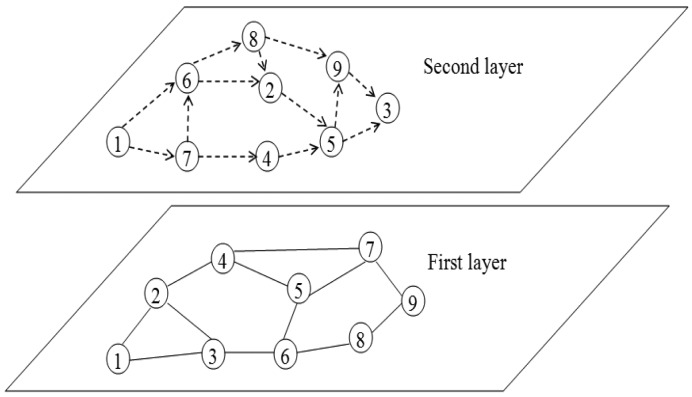
A sample two-layer architecture.

In order to realise the framework, a two-layer architecture is introduced, where the first layer is the wireless sensor network and the second layer is a multi-agent cooperation network, as shown in Figure 1.

In the first layer, the wireless sensor network layer, sensors are connected by a wireless medium, such as infrared devices or radio, which is represented by the solid lines connecting the two sensor nodes. The first layer is the physical network, which really exists. In the second layer, each sensor is modelled as an agent, and agents are linked by a cooperative relation, represented as dashed lines. In the rest of the paper, the two terms, sensor and agent, are used interchangeably. The second layer, the multi-agent cooperation network layer, is an abstract network, which does not really exist. The multi-agent cooperation network is formed on the basis of the sensors' past cooperation. For example, in Figure 1, if many packets sent from Sensor 1 have to be forwarded by Sensor 7, Sensor 1 may add Sensor 7 as one of its cooperative neighbours. Then, in the future, if Sensor 1 has packets to be sent, it first sends the packets to Sensor 7, and Sensor 7 then forwards the packets to the destinations for Sensor 1. For the process that Sensor 1 sends packets to Sensor 7 and Sensor 7 forwards packets to the destinations, Sensors 1 and 7 can use any existing routing approaches. It can be seen that the multi-agent cooperation network layer is used to guide the packet routing process in the wireless sensor network layer in order to improve the routing efficiency, instead of becoming a concrete routing protocol that directly operates in the wireless sensor network layer. Thus, in this case, many existing routing protocols can be employed in the wireless sensor network layer. Such a two-layer architecture is necessary, because in the first layer, the topology of a WSN cannot be altered (unless some nodes move or leave the network, or new nodes join the network), and hence, the proposed framework has to work in the second layer, the multi-agent cooperation network layer, which enables each agent to keep the most useful cooperative neighbours and, in turn, assists existing routing approaches to achieve better performance.

In this paper, the sensor network is defined as a graph, *G* = (*V,E*), where *V* = {*v*_1_,…,*v_n_*} is the set of *n* sensors in the network and *E* ⊆ *V* × *V* is a set of physical links. Two sensors, *v_i_* and *v_j_*, are physical neighbours if (*v_i_, v_j_*) ∈ *E* and they can directly transmit packets between each other. *N_i_* = {*v_j_*|(*v_i_*, *v_j_*) ∈ *E*} indicates sensor *v_i_'s* physical neighbour set. Similarly, the cooperation network is defined as a graph, as well, *G′* = (*A*, *E′*), where *A* is the set of agents and *E′* ⊆ *V* x *V* is a set of cooperation links. Each agent represents a sensor node. Two agents, *ag_i_* and *ag_j_*, are cooperative neighbours if (*ag_i_*, *ag_j_*) *E′*. ${N^{\prime }}_{i}=\left\{ag_{j}|\left(ag_{i},ag_{j}\right)\in E^{\prime }\right\}$ indicates agent *ag_i_*'s cooperative neighbour set. It should be noticed that physical neighbours are symmetrical, namely that if (v*_i_*, *v_j_*) ∈ *E*, then (*v_j_*, *v_i_*) ∈ *E* holds, but cooperative neighbours are asymmetrical, namely that when (*ag_i_*, *ag_j_*) ∈ *E′*, it is not necessary that (*ag_j_*, *ag_i_*) ∈ *E′* holds. Initially, each sensor's physical neighbour set is identical to its cooperative neighbour set (although later, its cooperative neighbour set may be adapted).

Before devising the framework, it is necessary to introduce some evaluation indices to estimate each sensor, as well as the sensor network. These indices include energy consumption, storage consumption and sensing coverage.

### 3.1. Energy Consumption

Energy consumption relies on how many packets are being transmitted and the transmission distance of each packet. For a single sensor, say *v_i_*, its energy consumption for transmitting a *k*-bit message to a receiver, say *v_j_*, with a distance *d* away can be calculated using Equation (1) [19].

(1) $${\text{Ene}}_{T_{i}}\left(k,d\right)=k\left(E_{\text{elec}}+\epsilon_{\text{amp}}d^{\beta }\right)$$

where *E_elec_* represents the energy being dissipated to operate the transmitter or receiver circuitry per bit and *ϵ_amp_* denotes the energy dissipation of the transmitter power amplifier for transmitting a bit to a receiver with a distance of *d* = 1 unit away, and *β* is a constant coefficient that denotes the path loss exponent.

For the receiver, *v_j_*, its energy consumption for receiving the *k*-bit message is:

(2) $${\text{Ene}}_{R_{j}}\left(k\right)=kE_{\text{elec}}$$

Thus, for the whole sensor network, its energy consumption in a pre-defined period is the sum of the energy consumption caused by sensor nodes transmitting and receiving data packets in that period.

Certainly, other factors, such as idle listening, overhearing and synchronisation, can also incur energy consumption. To simplify the model, such factors are not taken into consideration.

### 3.2. Storage Consumption

Storage consumption involves two types of consumption. The first type of storage consumption is for maintaining packets in sensors' waiting lists. For example, if a sensor has several packets to send, but in a time slot, it can only send one packet, it has to keep the remaining packets in its waiting list and transmit them in the succeeding time slots. It is important for a sensor to keep as few packets in its waiting list and as short of a period of time as possible, so that both the sensor's storage consumption and the packet transmission latency can be reduced. For a single sensor, *v_i_*, its storage consumption for maintaining packets can be calculated using Equation (3).

(3) $${\text{stor}}_{i}^{\left(1\right)}=S^{\left(1\right)}\cdot (\sum_{j=1}^{\left|W_{i}\right|}t_{ij}^{\left(1\right)})$$

where *W_i_* denotes the waiting list of sensor *v_i_*, $t_{ij}^{\left(1\right)}$ demonstrates the number of time slots during which sensor *v_i_* maintains a packet *j* and *S*^(1)^ is a constant to represent the storage coefficient for keeping packets.

The second type of storage consumption for an individual sensor is to keep (both physical and cooperative) neighbours, which can be calculated by employing Equation (4).

(4) $${\text{stor}}_{i}^{\left(2\right)}=S^{\left(2\right)}\cdot (\sum_{j=1}^{\left|N_{i}\right|}t_{ij}^{\left(2\right)}+\sum_{l=1}^{\left|{N^{\prime }}_{i}\right|}t_{il}^{\left(3\right)})$$

where *N_i_* and ${N^{\prime }}_{i}$ are the physical and cooperative neighbours of *v_i_*, respectively, which have been described above; $t_{ij}^{\left(2\right)}$ indicates the time slots during which sensor *v_i_* keeps *v_j_* as its physical neighbour; $t_{il}^{\left(3\right)}$ indicates the time slots during which sensor *v_i_, i.e.*, agent *ag_i_*, keeps *ag_l_* as its cooperative neighbour; *S*^(2)^ is a constant to represent the storage coefficient for keeping neighbours.

The reason for taking time into account when computing storage consumption is that in Equation (3), the time used to keep packets can indirectly reflect transmission latency, and in Equation (4), the time used to keep neighbours can, to some extent, reflect the computational overhead associated with taking each neighbour into consideration whenever a sensor sends a packet.

Likewise, for the whole sensor network, the storage consumption is the sum of each sensor's storage consumption.

### 3.3. Sensing Coverage

Sensing coverage is also an important property of a sensor network, although this property was overlooked by many researchers. As described in [21], saving unnecessary power consumption can prolong the lifetime of sensor nodes, but does not necessarily imply that a better sensing coverage can be achieved. Hence, it is necessary to consider sensing coverage as a separate property.

In many applications, sensor nodes are deployed randomly over the entire desired area. In this way, the sensing areas of different nodes may partially overlap. When a local area has a much higher node density than the average node density, a target location in that area may be covered by multiple sensors. On the other hand, if the node density of a local area is much lower than the average node density, a target location is more likely covered by only one sensor. The normalised effective sensing area, *η*, of a sensor, *v_i_*, is calculated using Equation (5) [21].

(5) $$\eta =\eta_{0}+\sum_{m=1}^{\infty }\frac{\eta_{m}}{m+1}$$

where *η*_0_ is the percentage of *v_i_*'s sensing area that is covered by *v_i_* only and *η_m_* represents the percentage of *v_i_*'s sensing area that is covered by *v_i_* and any other *m* neighbouring nodes. The value of *η* is within (0, 1]. If the sensing area of a node does not overlap with that of any other nodes, the value of *η* is one. On the other hand, if the sensing area of multiple nodes overlaps the sensing area of a node, the value of *η* may be much less than one.

For the whole sensor network, the sensing coverage is the ratio between the total sensing area covered by all sensor nodes and the target area. For example, if the target is 1000 m^2^ and all of the sensors totally cover 900 m^2^ (due to some sensors failure), the sensing coverage of this sensor network now is 0.9.

### 3.4. Framework Design

The aim of the multi-agent framework is to improve the overall routing performance of existing routing approaches. In this framework, each agent can autonomously create a cooperative neighbour set and each agent can dynamically add or remove cooperative neighbours into or from its cooperative neighbour set. Afterwards, when a sensor has a packet to send, it sends the packet to one of its cooperative neighbours, and the cooperative neighbour then relays the packet to one of the cooperative neighbour's cooperative neighbours until the packet reaches the sink node. For the packet transmission between cooperative neighbours, any existing routing approach can be used.

#### 3.4.1. Cooperative Neighbour Set Creation

The creation of a cooperative neighbour set is based on the sensors' historical information. We use an example to describe the creation procedure. A sensor, *v_j_*, often relays packets for *v_i_*. Then, *v_j_* may want itself to be *v_i_*'s cooperative neighbour. *v_j_* then sends a request message to *v_i_* to suggest *v_i_* add *v_j_* as one of *v_i_*'s cooperative neighbours. The request message includes the information regarding *v_j_* itself, such as the remaining energy of *v_j_*, the normalised effective sensing area of *v_j_*, the distance between *v_j_* to the sink node and the average number of hops from *v_i_* to *v_j_* (that is derived from the previously relayed packets, each of which contains a hop-count field). Once *v_i_* receives this request packet, *v_i_* will evaluate the reward of adding *v_j_* as one of *v_i_*'s cooperative neighbours. *v_i_* estimates that if it adds *v_j_* as a cooperative neighbour, *v_i_*'s storage consumption may increase and *v_j_*'s energy consumption may rise (because in the future, *v_i_* will send more packets to *v_j_* than before). Such a consumption is a negative reward. On the other hand, however, in the future, the packet routing delay could be reduced, because the routing procedure becomes more targeted. Furthermore, the energy consumption of other sensors could be reduced, since packets would be relayed by fewer sensors. The storage and energy consumption can be estimated using Equations (1), (2) and (4). The estimation of packet routing delay is based on two factors: the communication radius of a sensor, *r*, and the distance between sensor node *v_i_* and sink node *s*, which is recorded as *d_v_i_→s_*. If there is more than one sink in the sensor network, such a distance demonstrates the shortest distance. In this study, it is assumed that each sensor has the same communication radius, *r*. The distance between sensor nodes to the sink node can be obtained during the initialisation stage of a sensor network. Once the two factors, *r* and *d_v_i_→s_*, are known, the estimation of packet routing delay can be obtained using Equation (6), where *δ* is a constant coefficient, which is greater than one.

(6) $$\text{del}=\delta \cdot \frac{d_{v_{i}\to s}}{r}$$

The reason for using *r* and *d_v_i_→s_* to calculate the estimation of the packet routing delay is that the result of using *d_v_i_→s_* to divide *r* can approximately indicate the number of hops from sensor *v_i_* to sink *s*. Since a packet transmitted between two neighbouring sensors needs one time slot, the number of hops from sensor *v_i_* to sink *s* can approximately reflect the number of time slots needed to transmit a packet from *v_i_* to *s*, namely the packet delay from *v_i_* to *s*.

Hence, if *v_i_* wants to add *v_j_* as one of its cooperative neighbours, the estimated increase of storage consumption is $S^{\left(2\right)}\cdot {\hat{t}}_{ij}^{\left(3\right)}$, where ${\hat{t}}_{ij}^{\left(3\right)}$ indicates the intended time slots during which *v_i_* keeps *v_j_* as one of its cooperative neighbours; the estimated increase of energy consumption on *v_j_* is *ζ* · *Ene_Rj_* (*k*), where *k* denotes the total size of messages (measured in bits) passed from *v_i_* to *v_j_* and *ζ* is a constant coefficient, which is greater than one; the estimated routing delay reduction is *ζ* · (*del_i_* − *del_j_*); the estimated reduction of energy consumption on other sensor nodes is $\zeta \cdot {\text{hop}}_{i\to j}\cdot \left({\text{Ene}}_{T}\left(k,\frac{r}{\delta }\right)+{\text{Ene}}_{R}\left(k\right)\right)$. After the estimation, the reward of *v_i_*, namely 
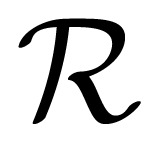
*_i_*, is derived by summing up the four estimated values. Here, the four estimated values can be assigned different weights in different situations to demonstrate their different importance levels. For example, if in some situations, energy consumption is the most important, the energy consumption will be assigned the highest weight among these estimated values. In this paper, we do not consider special use cases. Thus, the four estimated values are assigned the same weight, *i.e.*, one.

Once the reward value, 
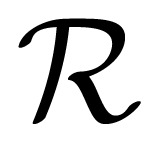
*_i_*, is obtained, sensor *v_i_* has to make a decision regarding whether to add *v_j_* as one of its cooperative neighbours. A Q-learning algorithm is developed for this decision making process (see Algorithm 1). Q-learning is one of the most-studied reinforcement learning (RL) algorithms and has been applied with success in several domains, from relatively simple toy problems, such as cliff-walking [30], to more complex ones, such as web-based education [31] and face recognition [32]. One of the attractive features of Q-learning is that it assumes no knowledge about the environment (such as state transition functions or reward functions).


**Algorithm 1.** Learning progress of sensor *v_i_*
**1**Initialise *Q* value for each available action arbitrarily;**2****for**
*k* = 0 to a predefined integer **do**;**3** calculate *π*;**4** **for each** available action *a* ∈ *A_i_*
**do**;**5**  *Q_k_*_+1_(*a*) = *Q_k_*(*a*) + *π* (*a*)*α*_1_(∑*_a_*

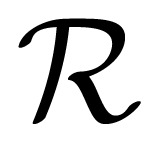
(*a*)*π*(*a*) − *Q_k_*(*a*));**6** **end for****7****end for****8***a_opti_* ← *argMax*(*Q*);**9***v_i_* takes the action *a_opti_*;


Sensor *v_i_* first arbitrarily initialises the Q-value of each available action (Line 1). There are two available actions for sensor *v_i_*: whether or not to add *v_j_* as one of its cooperative neighbours. Next, *v_i_* launches the learning process for the Q-value of each available action (Lines 2−7). In Line 2, the number of iterations is set to 30, which can yield adequate results. In Line 3, *π* is calculated using Equation (7) for each available action, *a*, which is the *ϵ*-greedy exploration method [33], where 0 < *ε* < 1 is a small positive number and *n* is the number of available actions of a sensor. The *ε*-greedy exploration method defines a semi-uniform probability distribution, in which the current best action is selected with probability 1 − *ϵ* and a random action is chosen with probability *ϵ*.

(7) $$\pi \left(a\right)=\{\begin{array}{l} \left(1-\epsilon \right)+\left(\epsilon /n\right),\text{if}\;Q(a)\;\text{is the highest value}\\ \epsilon /n,\text{otherwise} \end{array}$$

Line 5 demonstrates the Q-value update equation, where 0 < *α*_1_ < 1 is the learning rate and 
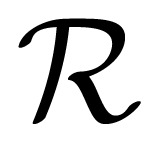
(*a*) is the estimated reward received by *v_i_* after taking action *a*. After finishing learning, *v_i_* executes the action that could maximise the Q-value (Lines 8–9). Finally, if *v_i_* decides to add *v_j_* as one of its cooperative neighbours, *v_i_* will send an acknowledgement message back to *v_j_* to let *v_j_* know that *v_j_* has been added to *v_i_*'s cooperative neighbour set. Conversely, if *v_i_* does not decide to add *v_j_* as its cooperative neighbour, *v_i_* just ignores *v_j_*'s request message and does not send any message back in order to save energy consumption. Having this algorithm, the expected behaviour of sensors can be derived by tracking the actions with the highest Q-value over the learning process of each sensor. The importance of obtaining a Q-learning algorithm with *ε*-greedy exploration is also justified through a large number of applications. For example, Galstyan *et al.* [34] applied a Q-learning algorithm with *ε*-greedy exploration to develop a decentralised resource allocation mechanism; Gomes and Kowalczyk [35] studied the problem of learning demand functions; and Ziogos *et al.* [36] investigated the development of bidding strategies.

Similarly, if *v_j_* has been a cooperative neighbour of *v_i_*, but *v_j_* seldom relays packets for *v_i_*, then *v_i_* may remove *v_j_* from its cooperative neighbour set so as to reduce *v_i_* computational overhead associated with taking *v_j_* into account whenever *v_i_* transfers a packet. The decision making of *v_i_*, regarding removing *v_j_* from its cooperative neighbour set, is analogous to the decision making process about adding *v_j_* as a cooperative neighbour.

Of course, a sensor can directly choose an action that could maximise its reward, and thus, the learning process is not really necessary. Kaelbling *et al.* [37] note, however, that this kind of algorithm, which always takes the highest rewards action and overlooks the trade-off between exploitation and exploration, may converge to a sub-optimal state.

#### 3.4.2. Selecting a Cooperative Neighbour for Relaying

When a sensor, say *v_i_*, has a packet to transfer, it needs to decide which cooperative neighbour is the best choice for relaying the packet. The selection of a cooperative neighbour is based on each cooperative neighbour's properties, which are embodied in the previous request message (As time progresses, the information embodied in the request message may be outdated. Thus, if a cooperative neighbour's status significantly changes, it will send a new message to *v_i_* regarding its current status.). These properties include the remaining energy of *v_j_*, the normalised effective sensing area of *v_j_*, the distance between *v_j_* to the sink node and the average number of hops from *v_i_* to *v_j_*. Generally, sensor *v_i_* prefers to forward packets to a cooperative neighbour, which has more remaining energy, because such a cooperative neighbour is not likely to run out of energy. *v_i_* also prefers a small normalised effective sensing area, because even if such a cooperative neighbour is depleted, the overall coverage area will not be greatly affected, since the sensing area of that cooperative neighbour is overlapped by multiple sensors. A short distance from *v_j_* to the sink and a small number of hops from *v_i_* to *v_j_* are also preferred, as a short distance and a small number of hops usually imply little transmission delay. Therefore, for *v_i_*, there are different benefits for choosing different cooperative neighbours to relay a packet. Such a benefit, for *v_i_* choosing *v_j_*, can be calculated using Equation (8).

(8) $$B\left(v_{j}\right)=\gamma_{1}\cdot {\text{Rem}}_{E}ng\left(v_{j}\right)-\gamma_{2}\cdot \eta \left(v_{j}\right)-\gamma_{3}\cdot d_{v_{j}\to s}-\gamma_{4}\cdot {\text{hop}}_{i\to j,}$$

where *Rem_E_ng*(*v_j_*) means the remaining energy of *v_j_, η*(*v_j_*) indicates the normalised effective sensing area of *v_j_, d_v_j__*_→_*_s_* is the distance from *v_j_* to the sink node, *hop_i_*_→_*_j_* denotes the number of hops from *v_i_* to *v_j_* and *γ*_1_, *γ*_2_, *γ*_3_, *γ*_4_ are coefficients that are used to normalise the four values in the same magnitude.

After calculation of the benefit for selecting each cooperative neighbour, sensor *v_i_* employs another Q-learning algorithm to select a cooperative neighbour to forward the packet (Algorithm 2).

In Line 1, *v_i_* initialises the Q-value of each cooperative neighbour as zero. ***π*** is a vector, which demonstrates the probability with regard to selecting each cooperative neighbour to transfer the packet, *i.e.*, $\pi =\langle \pi \left(v_{1}\right),\ldots ,\pi \left(v_{\left|{N^{\prime }}_{i}\right|}\right)\rangle$. *v_i_* initially sets each element in *π* to $\frac{1}{\left|{N^{\prime }}_{i}\right|}$ for simplicity. Then, in Lines 2−4, *v_i_* updates the Q-value of each cooperative neighbour based on the benefit of selecting each cooperative neighbour, 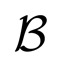
(*v_j_*), where α_2_ is the Q-learning rate. *v_i_* averages the benefits of selecting its cooperative neighbours and uses this average value and the newly created Q-values to update the probability vector *π*, where τ is the policy learning rate (Lines 5–8). The probability vector update method was developed by Zhang *et al.* [38]. In Line 9, the function *Normalise*() is used to constrain *π* to a legal probability distribution, *i.e.*, $\sum_{j=1}^{\left|{N^{\prime }}_{i}\right|}\pi \left(v_{j}\right)=1$, and *Normalise*(*π*) has the minimum Euclidean distance to *π*. Finally, in Line 10, *v_i_* selects a cooperative neighbour to transfer the packet based on the probability vector *π*. This selection is akin to the roulette, namely that cooperative neighbour *v_j_* with a large value of *π* (*v_j_*) is more likely to be selected. Algorithm 2 is a direct policy search algorithm that learns stochastic policies. As argued in [39], stochastic policies can work better than deterministic policies in partially observable environments (such as packet routing in WSNs, where each sensor has only partial information about the entire WSN). In this algorithm, the policy search is based on the difference between the benefit of an action and the current average benefit. Thus, this algorithm encourages each sensor to select the cooperative neighbours, which are associated with high benefits, for packet relaying. Since the benefit of selecting each cooperative neighbour is dynamic, a sensor does not always select relaying neighbours from a small part of its cooperative neighbours. Therefore, the relaying role can be automatically rotated among the sensor's cooperative neighbours, which can then prolong the lifetime of each cooperative neighbour.


**Algorithm 2.** Choice of a cooperative neighbour for relaying
\\ according to *v_i_'s* view \\**1**
*v_i_* initialises Q-values and *π*;**2 for each** cooperative neighbour of v*_i_, i.e.*, 
$v_{j}\in {N^{\prime }}_{i}$
**do****3** | *Q*(*v_j_*) ← (1 − *α*_2_)*Q*(v*_j_*) + *α*_2_ · 

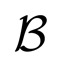
(*v_j_*);**4 end for****5**
$\bar{B}\leftarrow \frac{1}{\left|{N^{\prime }}_{i}\right|}\sum_{v_{j}\in {N^{\prime }}_{i}}B\left(v_{j}\right)$;**6 for each** cooperative neighbour of *v_i_*, *i.e.*, 
$v_{j}\in {N^{\prime }}_{i}$
**do****7** | *π*(*v_j_*) ← *π* (*v_j_*) + *τ*(*Q*(*v_j_*) − 

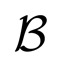
¯);**8 end for****9**
***π*** ← *Normalise*(*π*);**10**
*v_i_* selects a cooperative neighbour based on *π*;


In this framework, each sensor node in the network has to have a global unique ID to distinguish different nodes in the network (see Figure 1). Although some addressing schemes, e.g., [40,41], can be used to handle this problem, it is still sometimes difficult for each node to have a global unique ID, especially in a large WSN. This is a drawback of this framework, and overcoming this drawback will be the subject of one of our future studies.

### 3.5. Complexity Analysis of the Framework

This framework is proposed as an assistant tool to enhance the performance of existing routing approaches, but meanwhile, the framework incurs extra complexity, which is independent of the complexities of specific underlying routing approaches. Such complexity is incurred during the creation and use of cooperative neighbour sets. As described above, a node, e.g., *v_i_*, first estimates the reward of adding another node, e.g., *v_j_*, to *v_i_*'s cooperative neighbour set. Then, node *v_i_* uses Algorithm 1 to decide whether or not to add *v_j_* as one of its cooperative neighbours. In the future, when *v_i_* has a packet to send out, it will choose one of its cooperative neighbours to relay the packet.

The estimation of reward does not incur much extra complexity, as there is only the computation of simple equations (Equations (1)–(5)). Then, for the decision making of a node, in Algorithm 1, there is a nesting loop (Lines 2–7). The number of iterations of the loop, Lines 2–7, is 30. The number of iterations of the loop, Lines 4–6, is two, as there are two available actions: adding a node as a cooperative neighbour or not. Because the number of iterations of both of the loops is constant, the complexity of Algorithm 1 is 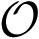
(1). For the selection of cooperative neighbours, in Algorithm 2, there are two loops. The number of iterations of each loop equals the number of cooperative neighbours of a sensor node. In the worst case, the number of cooperative neighbours of a sensor node is the number of sensor nodes in the WSN. As the number of sensor nodes in the WSN is *n*, the complexity of Algorithm 2 is 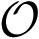
(*n*). It should be noted that to alleviate the potential worst case, sensor nodes are allowed to remove inefficient cooperative neighbours (recall Section 3.4.1).

In addition, there is also storage and communication consumption. Every time a node adds another node as a cooperative neighbour, the focal node has to use some storage space to store such a neighbouring relationship. In the worst case, a node adds all of the *n* nodes in the WSN to be its cooperative neighbours. Thus, the storage complexity is 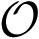
(*n*). Furthermore, before a node adds another node as a cooperative neighbour, a message has to be sent. Similarly, in the worst case, a node adds all of the *n* nodes in the WSN to be its cooperative neighbours, which means that *n* messages have to be sent. Thus, the communication complexity is 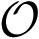
(*n*), as well. Certainly, the potential worst case can be alleviated by allowing sensor nodes to remove inefficient cooperative neighbours.

## 4. Simulation and Analysis

As illustrated in the previous section, the proposed framework is not a concrete routing approach, but rather a framework used to enhance existing routing approaches. Therefore, to assess the performance of the proposed framework, three existing routing approaches are selected, and each routing approach belongs to one of the three categories described in Section 2. Specifically, the selected routing approaches are gossiping [11] (a flat routing approach), LEACH-revised (a hierarchical routing approach) and GEAR [26] (a location-based routing approach). The reason for choosing the three routing approaches is that they are classical and easy to implement. Certainly, other routing approaches can also be employed in our simulation, but using the three classical approaches is sufficient to demonstrate the effectiveness of the proposed framework. Another of our future studies will take other more complex routing approaches into simulation. The three selected approaches are described in detail as follows.

1. Gossiping: In gossiping, a sensor node sends the packet to a randomly selected neighbour, which picks another random neighbour to forward the packet to, and so on, until the expiry time of the packet or the destination is reached, whichever comes first.
2. LEACH-revised: LEACH [19] randomly selects a pre-determined number of sensor nodes as cluster heads and rotates this role to evenly distribute the energy load among the sensors in the network. The role rotation is depicted as below. A sensor node chooses a random number, *r*, between zero and one. If this random number is less than a threshold value, *T*(*n*), the node becomes a cluster head for the current round. The threshold value is calculated based on an equation that incorporates the desired percentage, *p*, to become a cluster head in the current round and the set of nodes that have not been selected as a cluster head in the last $\frac{1}{p}$ rounds. Then, all elected cluster heads broadcast an advertisement message to the rest of the nodes in the network that they are the new cluster heads. The problem with LEACH, however, is that it assumes that the cluster heads can directly send packets to the sink node, which is infeasible in large-scale WSNs. Thus, we introduce Yu *et al.*'s multi-hop routing algorithm [42] used by cluster heads to transfer packets to the sink node. Yu *et al.*'s algorithm is based on the cost of each wireless link, which is evaluated by using the remaining power of each node.
3. GEAR: There are two phases in GEAR. First, forwarding packets toward the target region: upon receiving a packet, a node relays the packet to the neighbour which is closer to the target region than itself. If there is more than one neighbour, the nearest neighbour to the target region is selected. However, if all of the neighbours are further from the destination region than the node itself, one of the neighbours is picked based on their evaluated cost to the destination region. Second, forwarding packets within the region: if the packet has arrived at the target region, it is diffused in the region by either recursive geographic forwarding or restricted flooding, until the packet reaches the sink node.

### 4.1. Simulation Setup

The simulation is divided into two parts. One is in a stationary WSN, and the other is in a dynamic WSN. In the stationary WSN, the position of each sensor node, including sink nodes, is fixed, meaning that sensors cannot move, and the WSN is closed, which means that no new sensors will join the network. On the other hand, in the dynamic WSN, each sensor node, involving sink nodes, has a pre-defined probability to move, and the WSN is open, which means that at each time slot, with a pre-determined probability, a new sensor may join the network. Performance is measured by the four quantitative metrics: average delivery latency, successful delivery ratio, number of live nodes and total sensing coverage. The meanings of the four metrics can be easily deduced from their names. For packet delivery, it is supposed that a packet is transmitted and then received in one time slot. The simulation is operated in a simulated 1000 m × 1000 m area, and 200 sensor nodes and five sink nodes are deployed randomly in this area. The communication radius, that is the furthest delivery radius, of each sensor is 60 m, and the sensing coverage radius of each sensor is 40 m. The initial energy of each sensor is set to 5 J. The average size of a packet is 50 bytes, and the actual size of a packet is based on the normal distribution with variance equal to 10. At each time slot, each node generates a packet based on a pre-defined probability, and the expiry time of a packet is based on exponential distributions. If a packet cannot reach a sink before its expiry time, the delivery of this packet is considered as a failure. Undelivered packets are also accounted for in the average delivery latency computation. The values and meanings of those parameters mentioned in the previous section, are listed in Table 1. These values were chosen experimentally to provide the best results. Each simulation was executed 200-times, and the average results are displayed in the following figures. We used JAVA to build the simulation platform, where two two-dimensional arrays were used to represent the two layers of a sensor network and another two-dimensional array was defined to indicate the distance between two neighbouring sensors. ‘Sensor’ was programmed as a class, and each sensor is an object of this class.

### 4.2. Simulation Results: The Stationary Occasion

**Figure 2:**
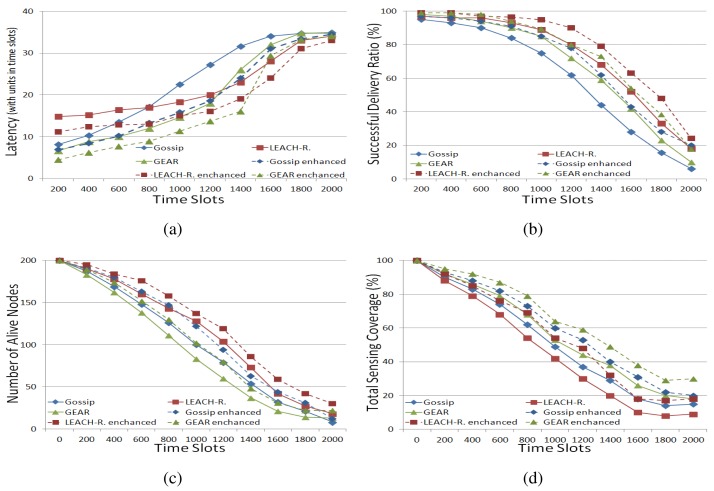
Performance of routing approaches in a stationary WSN. (**a**) Average delivery latency; (**b**) successful delivery ration (%); (**c**) number of live nodes; (**d**) total sensing coverage (%).

Figure 2 demonstrates the performance of the three routing approaches and their multi-agent framework-based enhanced versions in a stationary WSN. The solid lines denote the performance variation of the three routing approaches, while the dashed lines indicate the performance of their multi-agent framework-based counterparts.

In Figure 2a, initially, the delivery latency values in gossiping and GEAR are less than that in LEACH-revised. However, as time progresses, LEACH-revised turns out to be better than both gossiping and GEAR. This can be explained as follows. Initially, in LEACH-revised, the cluster heads usually have to wait for a period for their cluster members to send packets to them and then aggregate and condense these packets to save transmission energy consumption. As time progresses, some sensor nodes are depleted. In gossiping and GEAR, some pre-existing routing paths will disappear, but in LEACH-revised, the impact, caused by sensor node failure, is much less than that in gossiping and GEAR, because routing in LEACH-revised is based on only a few nodes, *i.e.*, cluster heads, and even if these cluster heads are exhausted, some other nodes can take their roles immediately by taking the measure developed in LEACH.

In Figure 2b, LEACH-revised achieved the highest successful delivery ratio. This is due to the fact that packets in LEACH-revised use the fewest hops from a source node to the sink node compared to gossiping and GEAR, because, as described above, routing in LEACH-revised is based only on cluster heads. Thus, it is more reliable for LEACH-revised to successfully transmit a packet to the sink node than both gossiping and GEAR.

In Figure 2c,d, it can be found that in gossiping and LEACH-revised, more sensor nodes can be saved than that in gossiping (Figure 2c), but in GEAR, the total sensing coverage is larger than that in both gossiping and LEACH-revised (Figure 2d). This finding confirms the argument in [21] that saving energy consumption can prolong the lifetime of sensors, but does not necessarily achieve better sensing coverage. This can be explained by the fact that GEAR's transmission method can easily deplete the nodes that are closer to the sink node, but gossiping's transmission method, randomly choosing a neighbour for transmission, can distribute the energy consumption over a larger area than GEAR's method does; so, in gossiping, fewer nodes are depleted compared to GEAR in the same time span. Likewise, in LEACH-revised, routing only relies on cluster heads, and the role of a cluster head is rotated among different sensor nodes. Thus, the lifetime of each sensor node in LEACH-revised could be further prolonged. However, due to the same reason, the total sensing coverage in GEAR is larger than that in gossiping, because gossiping's transmission method consumes energy over a larger area, which implies that depleted sensors are distributed over a larger area; so, the total sensing coverage reduction is more than that in GEAR. Similarly, in LEACH-revised, the energy dissipation in the network is distributed more broadly than it is in gossiping, because in LEACH-revised, sensors are organised based on clusters that are broadly distributed over the network. In this way, when sensors in different clusters are depleted, the total sensing coverage will be decreased more than that in gossiping. Finally, it can be seen that the performance of the three routing approaches is improved by the use of the proposed framework, measured by the four quantitative metrics. This is due to the fact that the framework enables each sensor to find the most useful cooperative neighbours, and therefore, the routing is more targeted. In addition, as described in the previous section, in the framework, each sensor takes into account energy consumption, storage consumption and sensing coverage, when it builds its cooperative neighbour set. Thus, by using the framework, the performance of the three existing routing approaches can be raised in all four quantitative metrics.

### 4.3. Simulation Results: The Dynamic Occasion

In this simulation, at each time slot, each sensor has the probability, 0.05, to move from its current position to a random position, and at each time slot, a new sensor will arrive at the network with the probability 0.2 at a random position. When an existing sensor moves to a new position or a new sensor joins the network, its physical neighbour set and cooperative neighbour set will be initialised. The node's physical neighbour set is built based on its communication radius. This means that any node in this node's communication radius will become this node's physical neighbours. The node's cooperative neighbour set is initialised in the same way as the physical neighbour set. In practice, the movement of a sensor is usually based on some purpose, so the sensor does not move to a random position. In this simulation, the aim is just to demonstrate how the proposed framework works in a dynamic WSN, so we simplified the movement of a sensor by setting its movement destination to a random position.

**Figure 3:**
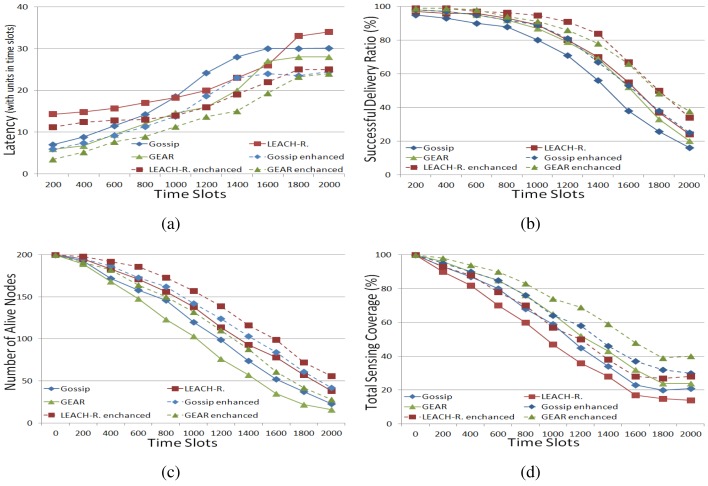
Performance of routing approaches in a dynamic WSN. (**a**) Average delivery latency; (**b**) successful delivery ration (%); (**c**) number of live nodes; (**d**) total sensing coverage (%).

In Figure 3, the main trend of the simulation results is similar to that in a stationary WSN, but the overall performance of the three routing approaches and their multi-agent framework-based enhanced versions is improved compared to that in a stationary WSN. This is because sensors movement can, to some extent, balance the energy consumption in the network. In a stationary WSN, those sensors that are neighbours of the sink nodes are very likely to be depleted, because they are the final hops to the sink nodes and all of the packets have to be relayed by them. Therefore, if sensors can move, those final hop nodes will most likely move away and, likewise, other sensors will most likely be the final hop nodes; so, the energy consumption can be balanced to some extent. Analogously, if new sensors can join the network, the energy of the whole network increases, which is good for the network in various aspects. There are still some specific phenomena that should be noted. In Figure 3a, the average delivery latency in LEACH-revised in the final time slots is longer (*i.e.*, worse) than that in gossiping and GEAR, which is different from Figure 2a. This is ascribed to the fact that routing in LEACH-revised is based on cluster heads, so new sensors joining or sensor movement cannot effectively shrink the routing path, and thus, the latency of packet routing cannot be improved very much. Due to the same reason, the difference of the final performance, regarding successful delivery ratio (Figure 3b) between LEACH-revised and gossiping is reduced compared to Figure 2b. On the other hand, in Figure 3c, the difference in the number of live nodes in LEACH-revised and gossiping in the final time slots is enlarged compared to Figure 2c, which can reflect the fact that LEACH-revised is quite good at balancing energy-consumption. Finally, again, using the proposed framework can improve the performance of all three routing approaches in a dynamic WSN.

In summary, using the proposed framework, the performance of the selected three routing approaches in both stationary and dynamic WNSs can be improved by around 10%–15% with regard to the four quantitative metrics, which demonstrates the potential usefulness of this framework.

## 5. Conclusions and Future Work

In this paper, a multi-agent framework was developed, which to the best of our knowledge, is the first one used to assist many existing routing approaches to improve their routing performance. In addition, this framework is decentralised and does not need global information. The simulation results exhibited the effectiveness of this framework in both static and open and dynamic WSNs.

In the future, we first intend to overcome the drawback that an addressing scheme has to be used. Another flaw is that this framework uses a ‘unit disk’ communication model, where sensors can communicate bi-directionally if they are in each other's communication radius. However, in real-world WSNs, several physical links are uni-directional. Additionally, more existing routing approaches have to be taken into our simulation to further validate the effectiveness of our framework, and it will be very interesting to allow nodes to use different routing approaches in different situations. We are also interested in validating the framework in heterogeneous WSNs, where different sensors have different sensing coverage and initial energy. Finally, when such studies are finished, we intend to test this framework on a specific platform, e.g., NS2 [43], and further in a real environment.

## Figures and Tables

**Table 1:** Parameter settings.

**Parameters**	**Values**	**Explanations**
*E_elec_*	50 *nJ/bit*	Energy dissipation parameter
*ϵ_amp_*	0.1 *nJ /bit/unit*	Energy dissipation parameter
*β*	3	Path loss exponent
***S***^(1)^	0.01	Storage coefficient for keeping packets
***S***^(2)^	0.0015	Storage coefficient for keeping neighbours
*δ*	2.5	Path length coefficient
ζ	2.2	Energy consumption coefficient
*α*_1_,*α*_2_,*τ*	0.1, 0.3, 0.2	Learning rate
*ε*	0.4	Action selection distribution probability
*k*	20	Learning rounds
*γ*_1_,*γ*_2_,*γ*_3_,*γ*_4_	1.7, 8.2, 0.013, 1.5	Benefit coefficients
